# Effects of vagus nerve stimulation on cognitive functioning in rats with cerebral ischemia reperfusion

**DOI:** 10.1186/s12967-016-0858-0

**Published:** 2016-04-27

**Authors:** Ai-fen Liu, Feng-bo Zhao, Jing Wang, Yi-Fan Lu, Jian Tian, Yin Zhao, Yan Gao, Xia-jun Hu, Xiao-yan Liu, Jie Tan, Yun-li Tian, Jing Shi

**Affiliations:** Department of Neurobiology, Tongji Medical College, Huazhong University of Science and Technology, Hangkong Road 13, Wuhan, 430030 Hubei China; Basic Medical Research Centre, Medical College of Nantong University, Nantong, 226001 Jiangsu China

**Keywords:** Vagus nerve stimulation, MCAO/R, DSP-4, Norepinephrine

## Abstract

**Background:**

Vagus nerve stimulation (VNS) has become the most common non-pharmacological treatment for intractable drug-resistant epilepsy. However, the contribution of VNS to neurological rehabilitation following stroke has not been thoroughly examined. Therefore, we investigated the specific role of acute VNS in the recovery of cognitive functioning and the possible mechanisms involved using a cerebral ischemia/reperfusion (I/R) injury model in rats.

**Methods:**

The I/R-related injury was modeled using occlusion and reperfusion of the middle cerebral artery (MCAO/R) in Sprague–Dawley rats. VNS was concurrently applied to the vagus nerve using a stimulation intensity of 1 mA at a fixed frequency of 20 Hz with a 0.4-ms bipolar pulse width. The stimulation duration and inter-train interval were both 3 s. Next, Morris water maze and shuttle-box behavioral experiments were conducted to assess the effects of VNS on the recovery of learning, memory, and inhibitory avoidance following I/R injury. Intracerebroventricular injection of N-(2-chloroethyl)-N-ethyl-2-bromobenzylamine hydrochloride (DSP-4), a selective neurotoxin for noradrenergic neurons, was used to evaluate the role of norepinephrine (NE) as a mediator of therapeutic effects of VNS on cognitive recovery.

**Results:**

Compared with the MCAO/R group, the VNS+MCAO/R group had improved spatial memory as indicated by swimming path lengths and escape latencies in the Morris water maze, and fear memory, as indicated by the avoidance conditioned response rate, mean shock duration, and avoidance time in shuttle-box behavior experiments. Compared with the VNS+MCAO/R group, the DSP-4+VNS+MCAO/R group, which had reduced NE levels in cortical and hippocampal brain regions, showed a reversal of the VNS-induced benefits on spatial and fear memory performance.

**Conclusions:**

VNS improves spatial and fear memory in a rat model of MCAO/R injury. However, a reduction in NE from the administration of DSP-4 blocks these protective effects, suggesting that NE may contribute to the influence exhibited by VNS on memory performance in rats with cerebral I/R-related injury.

## Background

Stroke is the leading cause of chronic adult disability and the third leading cause of death in the world [1–4]. Cerebral ischemia/reperfusion (I/R)-related injury can lead to brain damage, resulting in sensory and motor impairment [1–3, 5]. Although brain damage resulting from ischemia can be devastating, many patients survive the initial event and experience some degree of spontaneous recovery, which can be further augmented by rehabilitative therapy [2].

The aim of post-stroke recovery treatments is to enhance structural and functional reorganization (plasticity) of the affected brain areas [6, 7]. Methods such as repetitive transcranial magnetic stimulation and transcranial direct current stimulation (tDCS) have been identified as effective rehabilitative techniques [8, 9]. Although the efficacy of pharmacological techniques for enhancing post-stroke recovery from motor and cognitive impairments has not been conclusively demonstrated, there has been increased interest in pharmacotherapies that will potentially contribute to positive stroke rehabilitation outcomes [1]. Noradrenergic agonists have been the most extensively investigated pharmacological interventions. In animals with acquired brain injury, drugs that activate the noradrenergic system improve attention, responsiveness, and other cognitive skills [10]. Furthermore, medications that inhibit the noradrenergic system appear to have a negative influence on recovery. For example, administration of clonidine (an agonist of the α2-adrenergic receptor) impairs recovery of beam-walking after a sensorimotor cortex lesion in the rat [11]. Similarly, simultaneous administration of haloperidol (a α1-adrenergic antagonist) reverses the therapeutic effects of noradrenergic stimulation [12].

The vagus nerve is a major source of afferent information regarding visceral states, and provides input to the locus coeruleus (LC), which is the major source of norepinephrine (NE) in the brain [13, 14]. It has been hypothesized that release of NE contributes to the effects of electrical vagus nerve stimulation (VNS) on learning and memory, mood, seizure suppression, and recovery of function following brain damage. VNS increases extracellular NE concentrations in both the hippocampus and the cortex. However, the relationship between VNS and cognitive function needs to be further elucidated.

Therefore, we investigated the contribution of VNS to the recovery of learning and memory following I/R-related injury and the mechanisms involved in a rat model of occlusion and reperfusion of the middle cerebral artery (MCAO/R). The data demonstrate that VNS promotes recovery of spatial and fear memory in rats with ischemia-induced brain damage. We found evidence for the contribution of NE release induced by VNS. The present study contributes to the understanding of the effect of VNS on neuropsychiatric diseases and promotes discovery of novel treatments for ischemic brain injury.

## Methods

### Animals

Adult male Sprague–Dawley rats weighing approximately 250 g provided by the Center of Animal Experimentation of Tongji Medical College, Huazhong University of Science and Technology, China, were used in this experiment. Rats were housed with food and water ad libitum and a 12 h light/12 h dark cycle. Animal care was performed in accordance with guidelines approved by the US NIH and the Wayne State University Animal Investigation Committee.

### Rat MCAO/R model

**Fig. 1:**
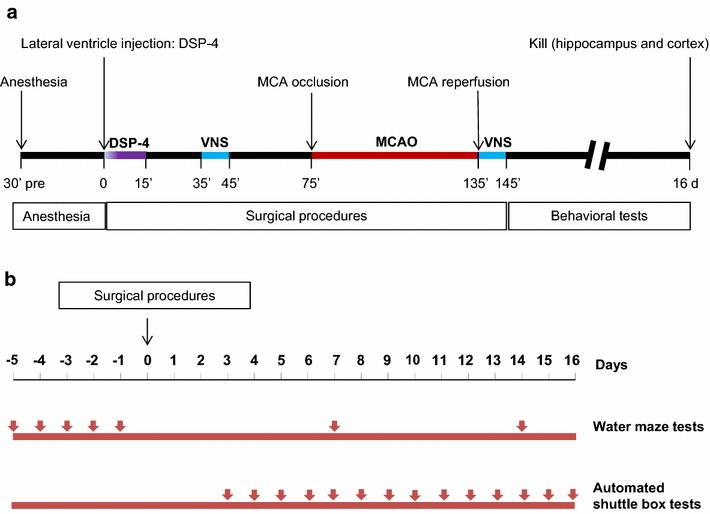
The experimental protocol. **a** The timeline of experimental procedures. After anesthesia smoothly, DSP-4 was administered intracerebroventricularly for 10 min after fully injected with flow rate 1 µl/min. Lateral ventricle injection time as a starting point. At 35 min, VNS was given just 30 min before MCA occlusion, and at the 135 min, VNS was again given just after MCA reperfusion. MCA: middle cerebral artery. VNS: vagus nerve stimulation. **b** The timeline of behavioral tests

Rats were anesthetized with 10 % hydration chlorine aldehyde (0.3 mL/kg intraperitoneal injections; i.p.). Briefly, cerebral ischemia was produced by intra-arterial filament occlusion of the left middle cerebral artery (MCA) for 1 h followed by reperfusion [6]. The left common carotid artery, external carotid artery (ECA), and internal carotid artery (ICA) were exposed. A length (18.5–19.5 mm) of 4–0 monofilament nylon suture (tip diameter 0.32–0.36 mm; Sunbio Biotech Limited Company, Beijing, China) determined by the weight of each rat, with its tip rounded by covering with Poly -l- lysine, was advanced from the ECA into the lumen of the ICA until it blocked the origin of the MCA. Reperfusion was performed by the withdrawal of the nylon suture 1 h after MCAO. The design of the experimental procedures is shown in Fig. 1.

### Vagus nerve stimulation

An incision approximately 2.0 cm in length was made on the left ventral side of the neck just lateral to the midline. The sternohyoid and sternomastoid muscles were separated longitudinally using small forceps and then retracted laterally until the carotid artery could be seen. The vagus nerve was then carefully separated from the surrounding connective tissue until a length of nerve sufficient for electrode placement was exposed. The left vagus nerve was then placed on a bipolar silver hook electrode, which was insulated from the surrounding tissue by a piece of soft plastic sheet. The two poles of the electrode were 5 mm apart. The stimulation intensity was 1 mA at a fixed frequency of 20 Hz with a 0.4 ms bipolar pulse width for 3 s and a 3-s inter-train interval. The stimulation protocol lasted for a total of 10 min. During the stimulation protocol, saxol was applied to the vagus nerve in order to prevent it from becoming dry. Middle cerebral artery occlusion was then performed as previously described. After 1 h of cerebral ischemia, the 10-min long VNS protocol was performed once more at the same intensity as the previous VNS. The incision area was then cleaned, antibiotic ointment was applied, and the incision was sutured. Rats were monitored until normal locomotion was observed, at which point they were returned to the housing room.

### Lateral ventricle administration

The selective NE neurotoxin N-(2-chloroethyl)-N-ethyl-2-bromobenzylamine-hydrochloride (DSP-4; 200 μg) was administered intracerebroventricularly to the left lateral ventricle (stereotaxic coordinates from skull surface: antero-posterior: +0.9 mm; medio-lateral: +1.4 mm; dorso-ventral: +3.4 mm). DSP-4were dissolved in 10 % saline containing 0.1 % ascorbic acid and the solutions were infused at 1 μL/min for 6 min.

### Water maze tests

The Morris water maze test was performed in a 1.25 m diameter circular water pool (Ethovision, Noldus Ltd, Wageningen, Netherlands) that was filled to a depth of 30 cm with 25 °C tap water. The water was mixed with an odorless non-toxic black dye. A black 10-cm-diameter columnar escape platform was placed in the water, positioned 1 cm below the surface of the water. Training consisted of four trials each afternoon for 5 consecutive days. During training, the platform was placed in a quadrant of the pool and the rat was placed in each of the 4 quadrants at the start the trial, positioned facing the wall. If the rat did not reach the hidden platform within 1 min, it was placed on the platform to rest for 30 s. The initial quadrant placement was consistent on each afternoon of testing. Latency to find the platform, path length, and swimming speed were measured for each rat.

### Automated shuttle box test

Experiments were conducted using fully automated 50 cm × 20 cm × 15 cm (length × height × width) shuttle boxes, equipped with a floor grid of 0.9-cm-diameter bars spaced 1.8 cm apart, and located in a sound-protected room. Loudspeakers were placed in the center of the ceiling above the boxes. Infrared light beams continuously determined each rat’s position and if they did not move to the opposite end of the box before the sound from the loudspeaker stopped, an electric shock was delivered from the floor grid, thereby incentivizing them to complete the task. In order to minimize odor cues, the shuttle boxes were cleaned with 75 % ethanol after each testing session. Each trial started with 2 min of habituation during which the rats were allowed to freely explore the shuttle boxes. Habituation was followed by learning trials where a conditioned stimulus consisting of a 2.4 kHz tone at 80 dB was presented for a maximum of 10 s. Next, the unconditioned stimulus, a 1.5 mA foot shock, was delivered for a maximum duration of 10 s. The inter-trial interval was 120 s and each rat completed 10 trials. The avoidance latency and the number of avoidance conditioned responses (CRs) were recorded and analyzed.

### Western blotting

In order to determine extracellular concentrations of NE following VNS and behavioral testing, rats were anesthetized, sacrificed, and the hippocampus and cortical brain tissues were harvested. Protein extracts of samples from the cortex and the hippocampus were prepared in RIPA buffer (Beyotime Institute of Biotechnology, Nantong, China) using standard methods. Thirty micrograms of total protein per lane was resolved by 10 % SDS-PAGE and transferred to polyvinylidene difluoride membranes. Normalization was performed using dopamine beta-hydroxylase (rabbit monoclonal; 1:1000; Epitomics Inc., Burlingame, CA, USA) and anti-β-actin (mouse monoclonal; 1:10 000; Abcam, Cambridge, UK) antibodies. Primary antibodies were detected using horseradish peroxidase-conjugated anti-rabbit or anti-mouse IgG and enhanced chemiluminescent reagent (ECL; Thermo-Pierce, Rockland, USA). Immunoreactive bands were detected using Kodak BioMax ML film. At least three independent replications were conducted for each experiment.

### Statistical analyses

All results are presented as mean ± SD. Two-way analysis of variance (ANOVA) and Bonferroni post hoc tests were performed for statistical comparisons between the groups. The two groups were compared using unpaired *t* tests. All results were considered significant at *p* < 0.05.

## Results

### Effects of VNS on spatial memory after cerebral I/R injury

**Fig. 2:**
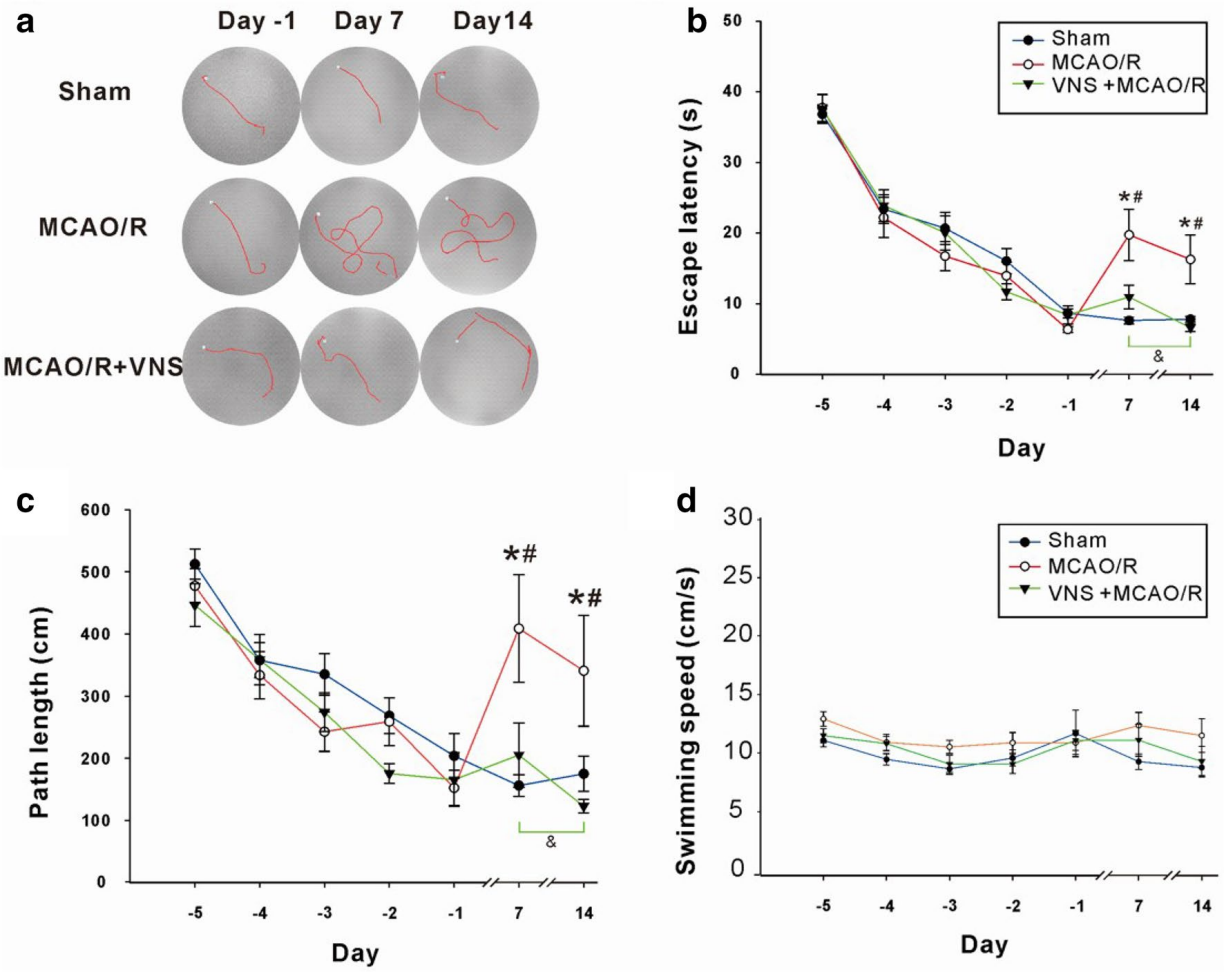
Vagus nerve stimulation (VNS) prevents memory loss after middle cerebral artery occlusion/reperfusion (MCAO/R) injury. **a** Typical traces from a water maze experiment recorded pre- (day-1) and post-surgery (day 7 and day 14), from the Sham (n = 12), MCAO/R (n = 11), and MCAO/R+VNS (n = 11) groups. Memory was assessed on days 7 and 14 after MCAO/R by measuring escape latencies (**b**), path lengths (**c**), and swimming speeds (**d**). *Indicates a significant difference between the MCAO/R and Sham groups. ^#^Indicates a significant difference between the MCAO/R and MCAO/R+VNS groups. &Indicates a significant difference between day 7 and day 14 (*p* < 0.05)

Rats were trained on the Morris water maze task for 5 d before surgery. On days 7 and 14 after surgery, animals were tested on the task to determine escape latencies (time to find the platform which was always on the same location at any time), path length, and swimming speed. On the swimming trajectory plots, the white box represents the starting place in the water tank, the red line represents the swim path, and the platform is positioned at the end of the red line (Fig. 2a). During the training trial, escape latencies and path length gradually decreased. For example, on the first day of training (day-5), the average escape latencies of the Sham, MCAO/R, and VNS+MCAO/R groups were 36.8, 37.7, and 37.6 s, respectively. However, on the fifth day of training, the escape latencies had decreased to 8.6, 6.4, and 8.4 s respectively (unpaired two-tailed *t* test: *t*(76) = 19.04, *p* < 0.0001 for Sham group; *t*(35) = 15.47, *p* < 0.0001 for MCAO/R group; *t*(44) = 12.31, *p* < 0.0001). Group differences at any point during the training were not significant. However, the escape latencies for the Sham group on days 7 and 14 after surgery were not significantly different from those of the later stages of training, whereas in the MCAO/R group, the post-surgery escape latencies increased to 19.7 and 16.3 s, respectively. In the VNS treatment group, the escape latencies were 10.9 and 6.6 s (escape latency was shorter on day 14 than on day 7), both of which were significantly lower than those in the MCAO/R group, but not significantly different from those in the Sham group (Fig. 1b) [two way ANOVA: *F* (2494) = 1.68, *p* = 0.1881, Bonferroni post hoc test: Sham vs. MCAO/R, *p* < 0.001 (day 7)]. The path lengths exhibited a similar trend in all three groups, decreasing gradually from an initial 500 cm to 200 cm at the end of training, without significant group differences. While changes in post-surgery path lengths exhibited a similar trend to those observed for escape latencies, path lengths were markedly longer on days 7 and 14 in the MCAO/R group (408.7 and 340.7 cm, respectively) than in the Sham group (156.1 and 151.4 cm, respectively). The path lengths were markedly shorter in the VNS treatment group (205.1 and 122.9 cm, *p* < 0.05) than in the MCAO/R group, but they were not significantly different from those of the Sham group [Two way ANOVA: *F* (2488) = 4.11, *p* = 0.017. Bonferroni post hoc tests: Sham vs. MCAO/R, *p* < 0.001 (day 7), *p* < 0.05 (day 14); MCAO/R vs. MCAO/R+VNS, *p* < 0.05 (days 7, 14) (Fig. 2c). Compared to pre-surgery results, there were no significant changes in swimming speed among any group [Two-way ANOVA, *F* (2, 150) = 6.97, *p* = 0.0013] (Fig. 2d). Thus, VNS effectively reduces spatial memory impairment after cerebral I/R-related injury in rats.

### Effects of VNS on impaired fear memory after cerebral I/R

**Fig. 3:**
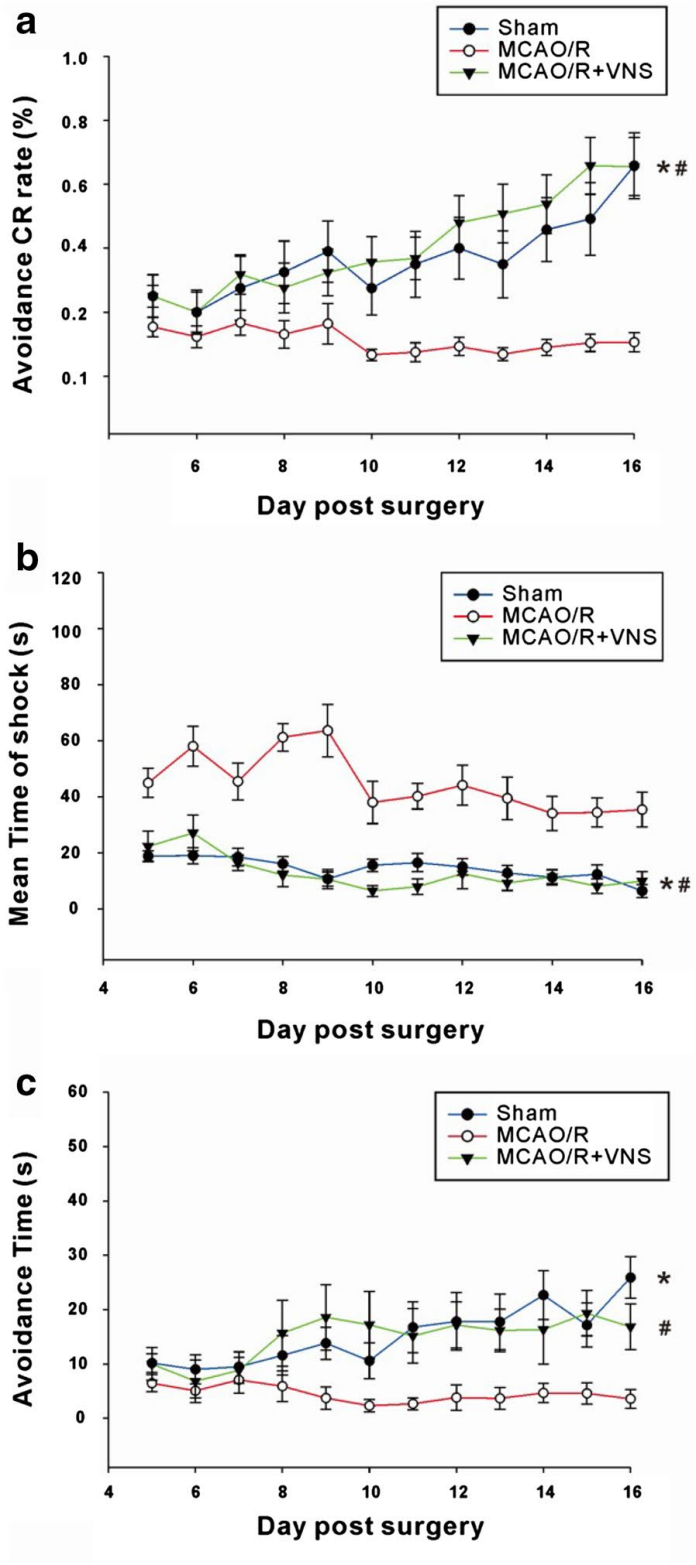
Vagus nerve stimulation (VNS) improves fear memory after middle cerebral artery occlusion and reperfusion (MCAO/R). From day 5 to day 16 post-surgery, rats in the Sham (n = 12), MCAO/R (n = 11), and MCAO/R+VNS (n = 6) groups were tested in the shuttle box and avoidance conditioned response rates (**a**), durations of shocks (**b**), and avoidance latencies were recorded (**c**). *,^#^Indicates significant differences (*p* < 0.05) between the MCAO/R and Sham groups and between the MCAO/R and MCAO/R+VNS groups, respectively

Changes in fear memory were examined using the shuttle box avoidance task on days from 5 to 16 after surgery. The number of electric shocks and avoidance responses were recorded on each testing day. The avoidance CR rate, mean shock duration, and latency to avoidance were calculated. As shown in Fig. 2, there was no initial difference in avoidance CR rates between the Sham, MCAO/R, and MCAO/R+VNS groups. For example, on post-surgery day 6, the avoidance CR rates were 20.0, 12.3, and 20.0 %, respectively. With increased training over time, the avoidance CR rates increased and reached 65.8 and 65.5 % at day 16 for the Sham and MCAO/R+VNS groups, respectively, whereas it remained low at 10.6 % in the MCAO/R group [Two-way ANOVA: *F* (2465) = 71.01, *p* < 0.0001. Bonferroni post hoc tests: sham vs. MCAO/R, *p* < 0.05 (days 11–13), *p* < 0.01 (day 14), *p* < 0.001 (days 15, 16); MCAO/R vs. MCAO/R+VNS, *p* < 0.05 (days 10–11), *p* < 0.001 (days 12–16)] (Fig. 3a). The mean shock duration was negatively associated with the avoidance CR rate for all groups. The mean shock duration had reduced in the Sham group from the initial 18.9 to 6.5 s, whereas in the MCAO/R group, the mean shock duration was markedly longer. The mean shock durations for the Sham group on post-surgery days 5 and 16 were 45.0 and 35.5 s, respectively with no significant group differences. In the VNS-treated rats, the mean shock duration was much shorter than that of the MCAO/R group, but was similar to that of the Sham group. Moreover, the 10-s mean shock duration on post-surgery day 16 was significantly shorter than the 22.3 s observed at day 5 [Two-way ANOVA, *F* (2315) = 165.78, *p* < 0.0001. Bonferroni post hoc tests: sham vs. MCAO/R, *p* < 0.01 (days 3–16); MCAO/R vs. MCAO/R+VNS, *p* < 0.05 (days 3–16)] (Fig. 3b). Avoidance latencies increased in the Sham group from 10.1 s on day 5 to 25.9 s on day 16. However, the avoidance latency of the MCAO/R group remained short at 6.5 and 3.6 s on days 5 and 16, respectively. The avoidance latency in the VNS group was between that of the Sham and MCAO/R groups and increased slowly from 10.0 s on day 5 to 16.8 s on day 16 [Two-way ANOVA, *F* (2323) = 42.73, *p* < 0.0001. Bonferroni post hoc tests: sham vs. MCAO/R, *p* < 0.01 (days 11–13); MCAO/R vs. MCAO/R+VNS, *p* < 0.05 (days 14, 16)] (Fig. 3c). These results indicate that VNS can effectively improve memory impairment in fear-conditioned animals after I/R-related injury.

### The effects of VNS and neurotoxin DSP-4 on NE levels in cortical and hippocampal brain regions

**Fig. 4:**
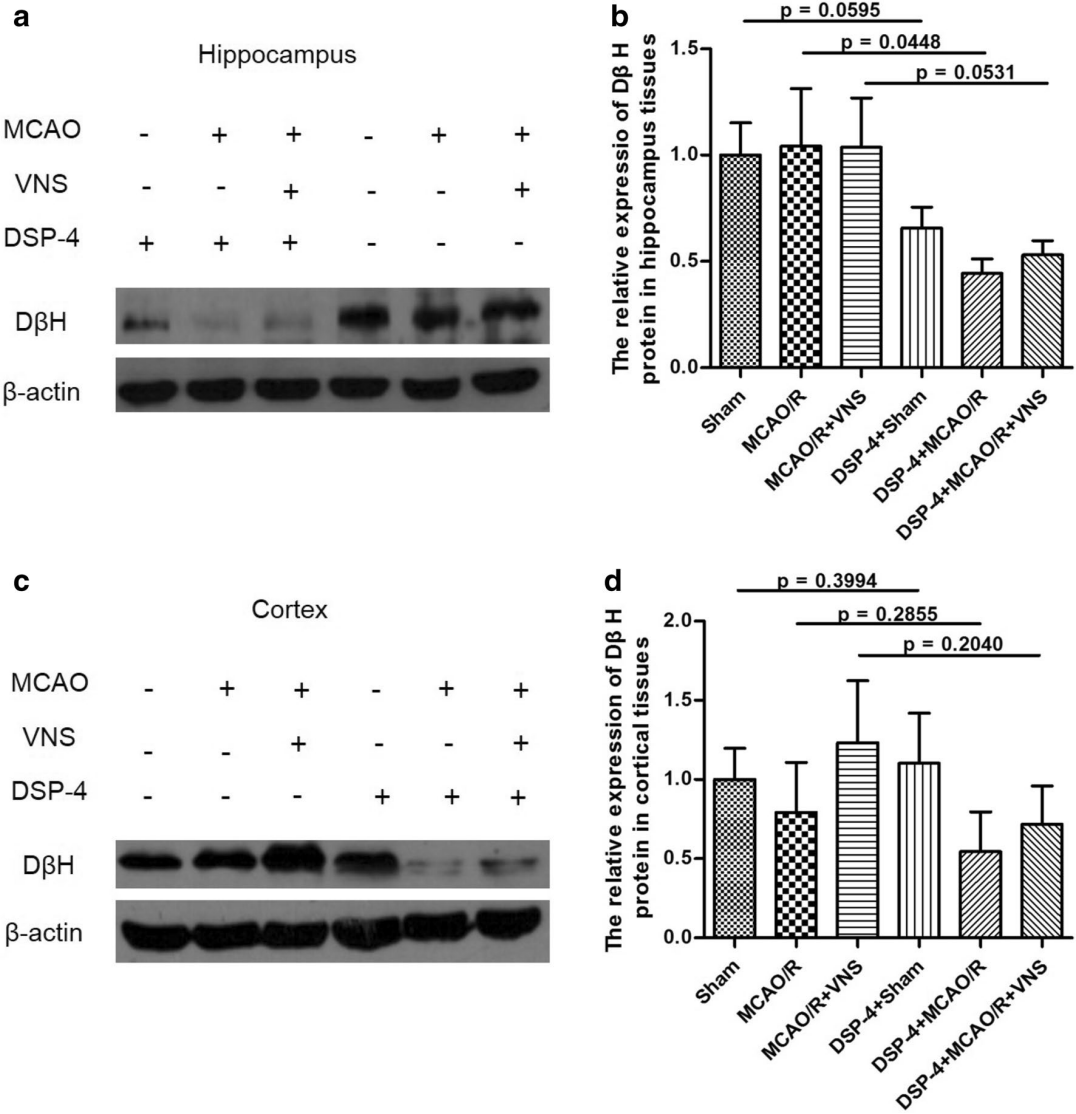
Effect of neurotoxin DSP-4 on dopamine beta-hydroxylase (DβH) levels after middle cerebral artery occlusion and reperfusion (MCAO/R). Neurotoxin DSP-4 inhibited the DβH levels in both hippocampal (**a**, **c**) (n = 11) and cortical (**b**, **d**) (n = 3) brain regions

Dopamine beta-hydroxylase (DβH), the enzyme that catalyzes the conversion of dopamine to norepinephrine, is released from sympathetic neurons. Therefore, in order to examine the effect of VNS and DSP-4 on NE levels in the cortical and hippocampal brain regions, we measured the expression of DβH using western blotting. Figure 4 shows that the DβH protein was inhibited by neurotoxin DSP-4.

### Damage to catecholaminergic neurons inhibited retention of the VNS-mediated effect on spatial memory

**Fig. 5:**
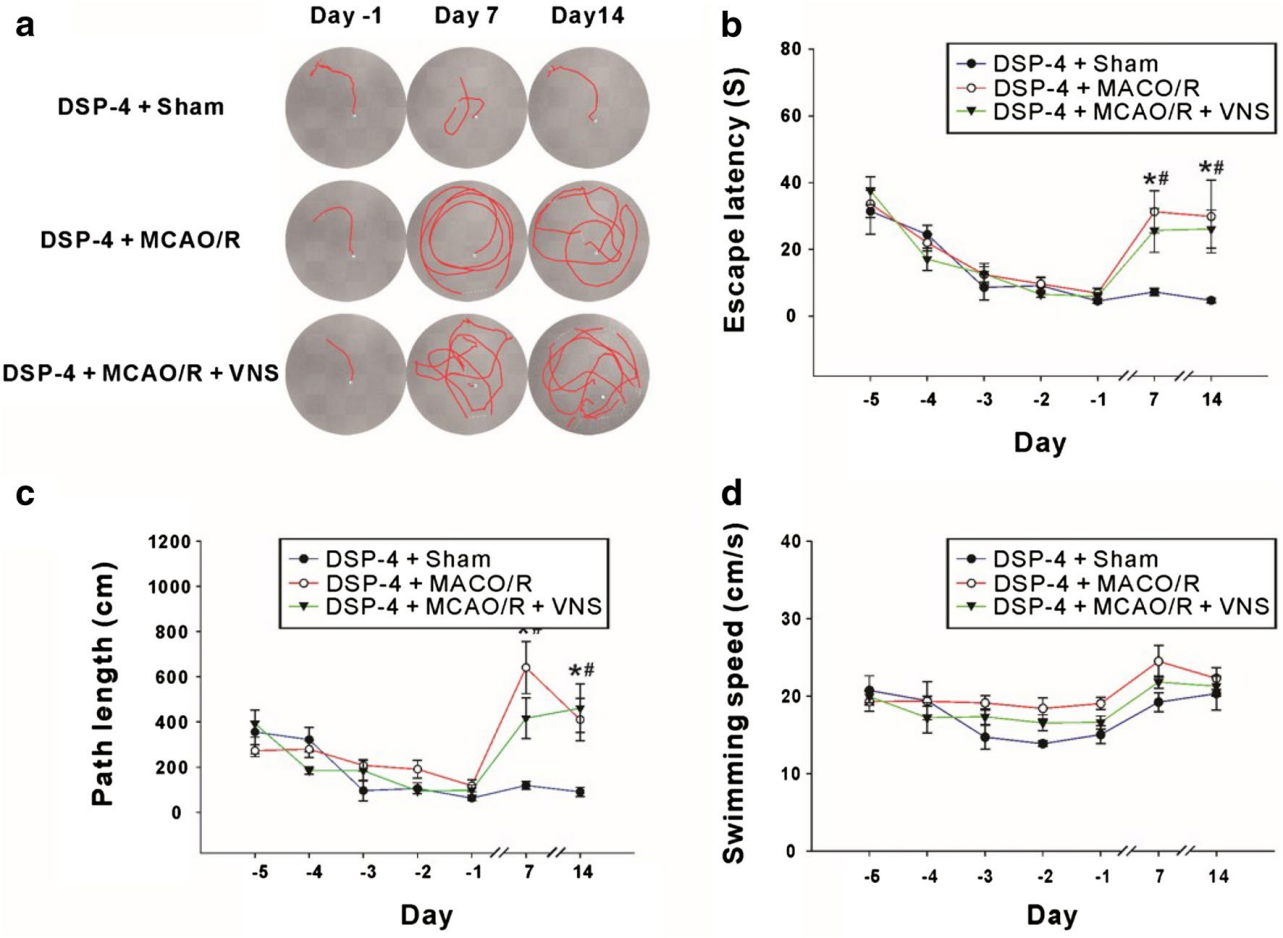
The role of vagus nerve stimulation (VNS) in spatial memory is blocked by norepinephrine depletion.** a** Typical traces of water maze activity on day-1, day 7, and day 14 relative to surgery were recorded from the DSP-4+Sham (n = 7), DSP-4+MCAO/R (n = 8), and DSP-4+MCAO/R + VNS (n = 8) groups. Escape latencies (**b**), path lengths (**c**), and swimming speeds (**d**) were observed during training (day-5 to day-1) and post-surgery (day 7 and day 14). *Indicates a significant difference between the DSP-4+MCAO/R and Sham groups, while ^#^Indicates a significant difference between DSP-4+MCAO/R+VNS and DSP-4+Sham groups. There was no difference between the DSP+MCAO/R and DSP-4+MCAO/R+VNS groups

The neurotoxin DSP-4, a chemical agent that damages noradrenergic neurons, was administered intraventricularly 30 min prior to surgery. Training procedures were performed as previously described and swimming trajectories were recorded on the Morris water maze task (Fig. 5a). As shown in Fig. 4, trained rats (day-1) could quickly locate the platform. On post-surgery day 7, the escape latencies of the DSP-4+MCAO/R group and the DSP-4+MCAO/R+VNS group were 640.3 and 416.6 s, respectively, which were significantly slower than the mean escape latency of the DSP-4+Sham group (119.5 s). Escape latencies did not significantly differ between the groups on post-surgery day 14 compared with those on post-surgery day 7 [Two-way ANOVA: *F* (2,140) = 7.61, *p* = 0.0007. Bonferroni post hoc tests: DSP-4+Sham vs. DSP-4+MCAO/R, *p* < 0.001 (days 7, 14); DSP-4+Sham vs. DSP-4+MCAO/R+VNS, *p* < 0.01 (day 7), *p* < 0.001 (day 14) (Fig. 5b). The swimming path length of rats in the DSP-4+Sham group was 63.1 cm on day-1 and 119.5 and 90.7 cm on post-surgery days 7 and 14, respectively. The swimming path length of the DSP-4+MCAO/R group increased from 117.8 cm before surgery to 640.3 cm and 410.27 cm on post-surgery days 7 and 14, respectively. The swimming path length of the DSP-4+MCAO/R+VNS group was similar to that of the DSP-4+MCAO/R group, and increased from 97.9 cm before surgery to 416.6 and 460.8 cm on post-surgery days 7 and 14, respectively. The swimming path lengths of the DSP-4+MCAO/R group and the DSP-4+MCAO/R+VNS group on post-surgery days 7 and 14 were not significantly different but were markedly longer than those in the DSP-4+Sham group [Two-way ANOVA: *F* (2,150) = 9.84,* p* < 0.0001. Bonferroni post hoc tests: DSP-4+Sham vs. DSP-4+MCAO/R, *p* < 0.001 (day 7), *p* < 0.01 (day 14); DSP-4+Sham vs. DSP-4+MCAO/R+VNS, *p* < 0.01 (day 7), *p* < 0.001 (day 14)] (Fig. 5c). No changes in swimming speeds occurred between pre- and post-surgery testing [Two-way ANOVA: *F* (2150) = 6.97, *p* < 0.0013. Bonferroni post hoc tests: *p* > 0.05.] (Fig. 5d). These results indicate that the previously observed protective effects of VNS on I/R-induced spatial memory impairment can be reversed by DSP-4, which damages noradrenergic neurons. Thus, VNS may exert its effects by increasing NE release.

### Damage to catecholaminergic neurons inhibits retention of the VNS-mediated effect on fear memory

**Fig. 6:**
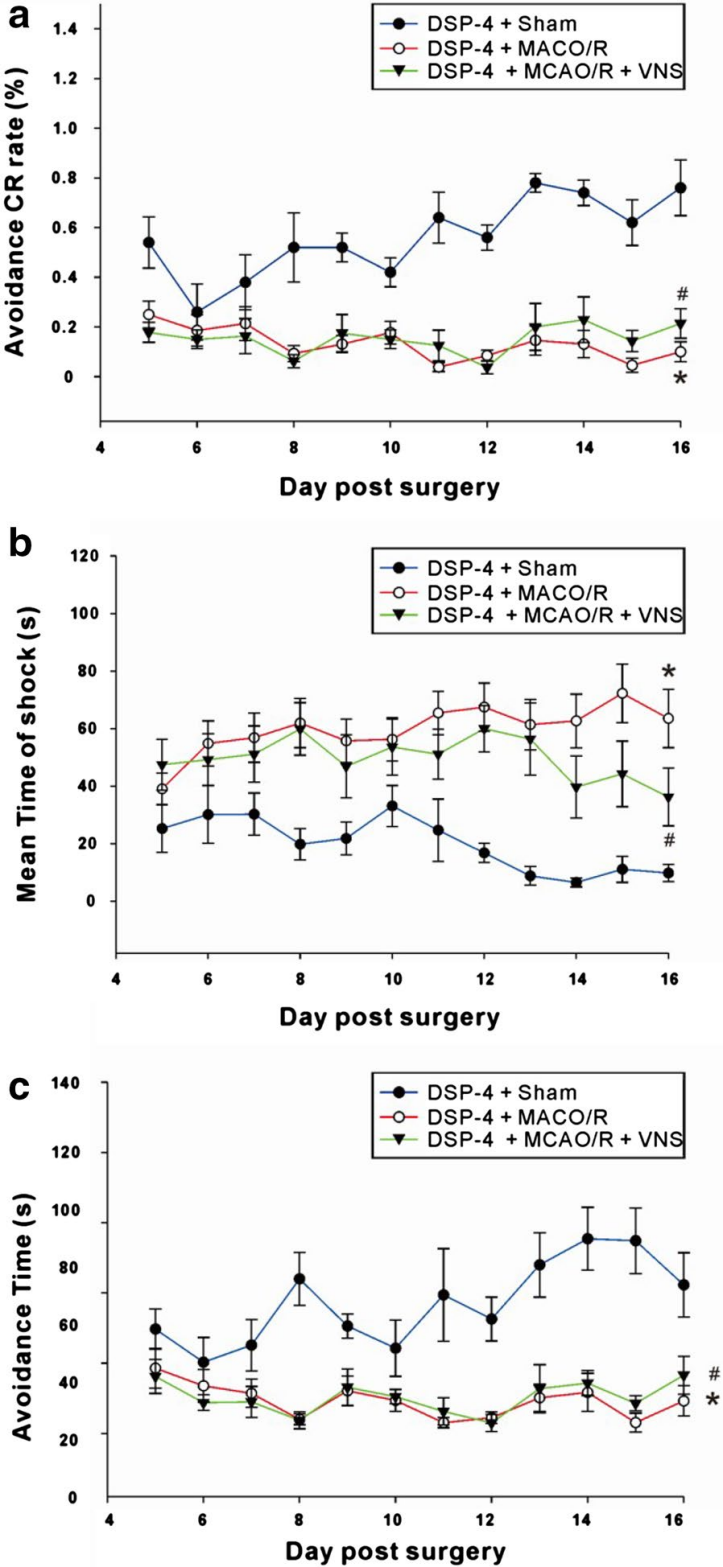
Vagus nerve stimulation (VNS) improves fear memory after middle cerebral artery occlusion and reperfusion (MCAO/R). From day 5 to day 16 post-surgery, rats in the DSP-4+Sham (n = 7), DSP-4+MCAO/R (n = 13), and DSP-4+MCAO/R+VNS (n = 8) groups were tested in shuttle boxes and the avoidance-conditioned response rates (**a**), duration of shocks (**b**), and avoidance latencies were recorded (**c**). *Indicates a significant difference between the DSP-4+MCAO/R and Sham groups and ^#^Indicates a significant difference between the DSP-4+MCAO/R+VNS and DSP-4+Sham groups. There was no difference between the DSP+MCAO/R and DSP-4+MCAO/R+VNS groups

Rats were treated intraventricularly with DSP-4 30 min before surgery and shuttle boxes were used to assess the number of electric shocks, mean shock duration, and avoidance latencies on post-surgery days 5–16. As shown in Fig. 5, the avoidance CR rate increased gradually with continued training in the DSP-4+Sham group. For example, the avoidance CR rate increased from 26.0 % on post-surgery day 6 to 76.0 % on post-surgery day 16. However, the avoidance CR rates did not improve with training in the DSP-4+MCAO/R group, yielding avoidance CR rates of 18.6 % and 10.0 % at post-surgery days 6 and 16, respectively. Moreover, the avoidance CR rates remained low in the DSP-4+MCAO/R+VNS group, at 15.0 and 21.4 % on post-surgery days 6 and 16, respectively [Two-way ANOVA: *F* (2324) = 71.01, *p* < 0.0001. Bonferroni post hoc tests: DSP-4+sham vs. DSP-4+MCAO/R, *p* < 0.01 (day 8, days 13–14), *p* < 0.05 (day 16)] (Fig. 6a). For the mean shock duration in the DSP-4+Sham group, the initial rate diminished from 30.1 to 9.9 % on post-surgery day 16. In the DSP-4+MCAO/R group, the mean shock durations were 54.9 and 63.5 % on days 6 and 16, respectively, with no significant differences between pre- and post-training rates. In the DSP-4+MCAO/R+VNS group, the mean shock durations did not differ significantly and were 36.3 and 49.3 % on days 6 and 16, respectively. Although the mean shock duration in the DSP-4+MCAO/R+VNS group was slightly lower than that in the DSP-4+MCAO/R group on post-surgery day 16, it was substantially higher than that in the DSP-4+Sham group [Two-way ANOVA: *F* (2299) = 61, *p* < 0.0001. Bonferroni post hoc tests: DSP-4+Sham vs. DSP-4+MCAO/R, *p* < 0.05 (day 11), *p* < 0.01 (day 8), *p* < 0.001 (days 12–16); DSP-4+Sham vs. DSP-4+MCAO/R+VNS, *p* < 0.05 (days 12, 16), *p* < 0.01 (day 13)] (Fig. 6b). Avoidance latency in the DSP-4+Sham group gradually increased with continued training, from 10.1 % on day 6 to 21.1 % on day 16. In contrast, no change occurred between pre- and post-training in the DSP-4+MCAO/R group, with avoidance durations of 6.8 and 4.6 % on days 6 and 16, respectively. The avoidance latency for the DSP-4+MCAO/R+VNS group was not significantly different from that of the DSP-4+MCAO/R group, at 4.4 and 8.3 % at days 6 and 16, respectively [Two-way ANOVA: *F* (2298) = 96.98, *p* < 0.0001. Bonferroni post hoc tests: DSP-4+Sham vs. DSP-4+MCAO/R, *p* < 0.01 (day 12), *p* < 0.001 (days 8, 11, 13, 14, 15); DSP-4+Sham vs. DSP-4+MCAO/R+VNS, *p* < 0.05 (day 16), *p* < 0.001 (days 8, 11, 13, 14, 15)] (Fig. 6c). There was no difference between pre- and post- training values for the DSP-4+MCAO/R group (Fig. 6c). These results indicate that the VNS-mediated improvement in the I/R-induced behavioral impairment in fear-conditioned rats can be inhibited by treatment with DSP-4, providing further evidence that NE may mediate the effects of VNS.

## Discussion

VNS has been widely used clinically for treating drug-resistant depression and refractory epilepsy. Recently, it was reported that VNS reduced infarct size in a rat model of cerebral I/R. However, the use of VNS as a treatment for cerebral I/R-related injury has not been thoroughly investigated. Using the Morris water maze and shuttle box behavioral tests, we found that VNS promoted a cognitive recovery that was reversed following the administration of neurotoxin DSP-4, suggesting that the benefits of VNS are mediated by NE.

The afferent vagal fibers that enter the nucleus of the solitary tract (NTS) have widespread projections in the brain stem and the forebrain and subsequently project directly and indirectly to the LC [15]. Research findings indicate that VNS activates neurons in the LC. For example, electrical stimulation of the vagus nerve in the rat increases the discharge rate of LC neurons [16]. LC neurons project to the hippocampus via the dorsal bundle and are the main source of NE in the hippocampus [17]. In addition, the LC is the primary source of NE in the ipsilateral cortex [18]. Therefore, VNS likely exerts its effects by stimulating noradrenergic neurons of the LC to release NE on downstream targets such as the cortex, hippocampus, and amygdala. In general, increasing the effects of NE at synapses can improve recovery from brain damage [19] and NE reuptake inhibitors likewise improve functional recovery after stroke in humans [20]. In contrast, NE antagonists impair recovery after a sensorimotor cortex lesion in rats and may even result in a regression to the original, post-injury state [11]. Characteristics of the animals, particularly the state of arousal, can modulate performance on a variety of learning and memory tasks including escape suppression, navigation through spaces, and verbal memory [2, 21]. These effects are attenuated by vagotomy, drugs that block arousal, or NTS damage. Furthermore, electrical stimulation of the vagus nerve improves performance on memory tasks in rats [22] and humans [23].

Electrical stimulation of cervical vagal afferent fibers is a safe and effective treatment for refractory epilepsy and drug-resistant depression in clinical settings [24]. It is also a potential treatment for migraine, senile dementia, traumatic brain injury, neuropathic pain, and Alzheimer’s disease and is currently being evaluated as such in on-going clinical studies [24–27]. Recently, VNS has been reported to provide protection against cerebral ischemic injury in the rat [28–31]. VNS regulates several cerebral I/R-related pathways and inhibits cytokine synthesis, thereby preventing I/R-related cytokine-mediated tissue damage [32]. In addition, VNS is associated with reduced neuronal excitability [33]. In 5 min after ischemia in a gerbil model for VNS reduced hippocampal damage by 50 % [34]; In a rat model of focal cerebral ischemia, VNS reduced infarct size [30]. Therefore, VNS is an efficacious neuroprotective agent against acute cerebral ischemic injury. VNS may improve treatment for cerebral ischemic damage and promote rehabilitation after injury by modulating endogenous brain activity.

In the present surgical procedures, we selected the left vague nerve stimulation, which is unlikely to lead to an impairment in cardiovascular functioning. First, vagal innervation of the heart is asymmetric as there is very little innervation of the heart by the left vagus nerve while the AV node is the primary region of innervation. In contrast, the right vagus nerve innervates the sinus node (i.e., the primary pacemaker) and atriums. Thus, the left VNS exerts little effect on heart rate. Second, when downstream efferent fibers are blocked by lidocaine, the influence of VNS on behavior remains. Third, NE release still occurs when the peripheral influence of VNS is blocked by atropine [35]. Therefore, this evidence indicates that the influence of VNS on the central nervous system is independent from its cardiovascular effects.

The outcome of VNS treatment is closely related to its specific parameter settings. Different stimulation parameters such as current intensity, pulse width, frequency, and cycle switching can induce varying types or levels of neurotransmitter release in different brain regions. Previous research has confirmed that a paucity of behavioral changes were observed with 0.25 mA stimulation of the vagus nerve, but 0.5 mA and 1 mA stimulations produced simple effects on the respiratory form, such as changes in rhythm. Trembling or increased neck muscle tension was also sometimes observed with these parameters [36]. Previous studies also found that the intensity of the electrical current used for VNS stimulation is closely associated with the level of NE release. VNS increases the concentration of NE in the hippocampus in a magnitude-dependent manner and the difference in responding to 0.5 and 1 mA VNS is substantial. However, in the cortex, only 1 mA VNS significantly increases the concentration of NE. In the cortex and hippocampus, VNS-induced increases in NE are transient with stimulus-induced elevations and return to baseline levels in the inter-stimulus period [37]. The VNS-induced increase in NE is therapeutically beneficial. Studies have reported on the effects of VNS administration on depression and epilepsy that provide reference for selecting stimulation parameters. Nevertheless, systematic exploration of the most appropriate combination of stimulation parameters is important for individualized therapy, improvements in efficacy, the reduction of side effects, and other considerations in the use of VNS. Collectively, the acute stimulation parameters that were applied in our present studies were 1 mA intensity, 20 Hz frequency, bidirectional pulse with 0.4 ms pulse width, 3-s intervals after 3 s of stimulation, and 10 min total stimulation for the duration of the protocol.

In the present study, VNS was applied 30 min before and after ischemia as an acute single protocol. However, VNS-induced improvements in cognitive functioning occurred from several days to several weeks after I/R-related injury, indicating that the effects of VNS are more likely to initiate protective and repairing functions of the body rather than to maintain these functions. Neurons in the penumbra of cerebral I/R injury have two possible fates: survival or death. Effective interventions made within hours after injury are important prognostic factors in outcome. The time window for acute VNS treatment is closely related to the death or survival of neurons in ischemic penumbra. Therefore, the most likely mechanism by which acute VNS exerts its effect is protection. Acute VNS may also induce the expression and activation of neurotrophic factors such as brain-derived neurotrophic factor (BDNF) and enhance the plasticity of surviving neurons. Regardless, both mechanisms require further experimental investigation.

In order to further define the molecular mechanisms by which VNS improves cognitive functioning after ischemia, the noradrenergic neurotoxin DSP-4 was administered intraventricularly and the role of NE in cognitive functions such as spatial working memory was evaluated. This rat model of brain damage specifically targets noradrenergic projections originating from the LC and dose-dependently reduces NE levels in the brain [38]. Studies have shown that the release of the sympathetic neurotransmitter norepinephrine occurs by exocytosis in which the vesicular contents of the soluble protein DßH are also released [39] and therefore the presence of this protein was used to estimate the release of NE in the hippocampus and cortex in the present study. DSP-4 is an alkylating agent that forms covalent bonds with electrophilic centers and is transported to the presynaptic membrane by high affinity interactions with the system that inactivates NE neurons [40]. The effects of DSP-4 are associated with the number of [3H] nisoxetine binding sites, but not the affinity for them. DSP-4 at concentrations of 10, 20, 50 and l00 mg/kg can dose-dependently reduce cortical NE by 51, 73, and 100 %, respectively, while concentrations of monoamines such as 5-HT and dopamine do not change [38]. Therefore, in the present study, we used 100 mg/kg of DSP-4 to damage noradrenergic neurons in the LC and observed the reversal of the therapeutic effects of VNS. Our findings indicate that VNS may exert its effects via NE release from noradrenergic neurons in the LC. Intraventricular administration of DSP-4 had no effect on cognitive behavior in sham-operated control rats and minimal effects on rats with I/R-related injury. However, there were prominent cognitive effects of DSP-4 in rats treated with VNS after I/R. DSP-4 almost completely inhibits the protective and restorative effects on cognitive function associated with VNS treatment.

In the present study, spatial memories and fear memories were considered behavioral indicators of the functional integrity of the hippocampus and cortex using the water maze task and automated shuttle box test, respectively. VNS treatment significantly improved impairments in spatial and fear memory in MCAO/R group, which was reversed by administration of DSP-4. Previous studies have shown that the hippocampus and infralimbic cortex are involved in memory deficits observed following I/R-related injury. Therefore, the results of this study suggest that VNS attenuates memory impairments following I/R-related injury by projecting neurons and releasing NE to the hippocampus and infralimbic cortex through the LC nucleus.

Inevitably, there were a few limitations to our experimental design. For example, we did not include a group treated with VNS alone and therefore, the improved behavioral performance could be attributable to VNS itself and not the reversal of the MCAO/R effect. In addition, the timing of VNS was selected for suitability in possible future clinical applications. Despite these limitations, the present study provides a potential treatment strategy for cerebral I/R-related injury.

## Conclusions

VNS stimulation significantly improves spatial and fear memory after cerebral ischemia in rats. The therapeutic effects of VNS are associated with NE release. Although few studies have investigated VNS as a treatment following cerebral I/R, recent findings indicate that it is a promising option. The present study also contributes to the understanding of the effects of VNS on neuropsychiatric diseases and promotes discovery of novel strategies for treating ischemic brain damage.

## Abbreviations

VNS: vagus nerve stimulation
I/R: ischemia/reperfusion
MCAO/R: occlusion and reperfusion of the middle cerebral artery
NE: norepinephrine
tDCS: transcranial direct current stimulation
CNS: central nervous system
LC: locus coeruleus
ECA: external carotid artery
ICA: internal carotid artery
MCA: middle cerebral artery
CR: conditioned response
DβH: dopamine beta-hydroxylase
CNS: central nervous system
LC: locus coeruleus
ECA: external carotid artery
ICA: internal carotid artery
MCA: middle cerebral artery
CR: conditioned response
DβH: dopamine beta-hydroxylase
